# Structural Organization of Imidazolium-Based Ionic Liquids and Metal–Organic Frameworks within a Fluorinated Polymer Matrix and Its Influence on the Physical-Chemical Response

**DOI:** 10.1021/acsapm.5c02138

**Published:** 2025-09-05

**Authors:** Liliana C. Fernandes, Bruna F. Gonçalves, Viktor Petrenko, Leide P. Cavalcanti, Carmen R. Tubio, Gabriela Botelho, Carlos M. Costa, Daniela M. Correia, Senentxu Lanceros-Mendez

**Affiliations:** † Physics Centre of Minho and Porto Universities (CF-UM-UP) and Laboratory of Physics for Materials and Emergent Technologies LapMET, 56059 University of Minho, Braga 4710-053, Portugal; ‡ 518636 BCMaterials - Basque Center for Materials, Applications, and Nanostructures UPV/EHU Science Park, Leioa 48940, Spain; § Ikerbasque, Basque Foundation for Science, Bilbao 48009, Spain; ∥ 120797 ISIS Neutron and Muon Source, Science and Technology Facilities Council, Rutherford Appleton Laboratory, Didcot OX11 0QX, U.K.; ⊥ Centre of Chemistry, 56059 University of Minho, Braga 4710-053, Portugal

**Keywords:** Ionic liquids, metal−organic frameworks, nanoscale structural organization, fluorinated matrix, PVDF, SANS

## Abstract

Ionic liquids (ILs) and metal–organic frameworks (MOFs) have been combined among themselves and with polymer matrices to obtain composites that are able to be applied in different fields, ranging from sensors and actuators to energy devices. Besides the great efforts devoted to the development and application of those composites, detailed information regarding the internal organization of IL and MOF within the composites is still needed, in order to further tune material properties toward applications. Thus, the present work reports on the development of polymer composites based on the MOF Basolite C300-HKUST-1 and different imidazolium-based ILs ([Bmim]­[FeCl_4_], [Bmim]­[N­(CN)_2_], and [Bmim]­[SCN]) with different types of anions incorporated into a fluorinated poly­(vinylidene fluoride) (PVDF) polymer matrix. Ternary MOF/IL/PVDF composites have been prepared by solvent casting incorporating 20 wt % of the different ILs together with 20 wt % of HKUST. The influence of MOF and ILs into the morphological, physical-chemical and electrical properties of the composites was evaluated. No significant morphological differences were observed upon the incorporation of the different ILs and MOF into the PVDF, all displaying compact microstructures. However, independently of the IL type, an increase in the electroactive β phase of the polymer was observed, with the highest amount in the ternary composite containing the IL [Bmim]­[N­(CN)_2_], reaching approximately 74%. Furthermore, the degree of crystallinity of the composites increased with the IL incorporation. The electrical properties of the different ternary composites revealed an increase in the conductivity from 1.06 × 10^–13^ S·cm^–1^ for the pristine polymer to 7.48 × 10^–9^ S·cm^–1^, 7.52 × 10^–7^ S·cm^–1^, and 3.11 × 10^–9^ S·cm^–1^ for the ILs [Bmim]­[FeCl_4_], [Bmim]­[N­(CN)_2_], and [Bmim]­[SCN]-containing samples, respectively. The internal structure of the composite samples was evaluated, showing that the presence of [Bmim]­[SCN] and [Bmim]­[FeCl_4_] in the ternary composites leads to a destruction of the MOF structure as well as to large-size inhomogeneities, indicating that IL accumulated with parts of the destroyed MOF as well as IL agglomeration through the matrix. These findings demonstrate the ability to tailor the electroactive phase content and conductivity through IL selection, which is highly relevant for functional applications. In particular, the developed composites hold strong potential for use in flexible sensors, electrochemical energy storage systems, and responsive devices where ionic conductivity and structural organization at the nanoscale are relevant.

## Graphical abstract

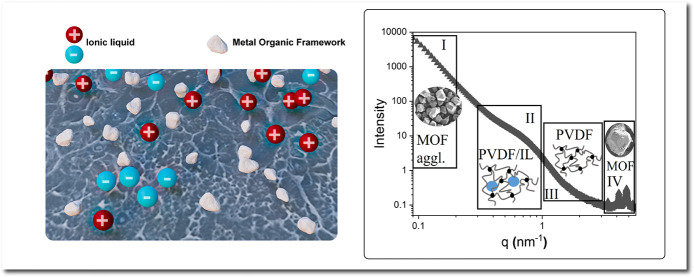

## 1 Introduction

Since ionic liquids (ILs) emerged, they have been demonstrating their strong potential in several application areas including batteries, tissue engineering, sensors and actuators, fuel cells, and catalysis, among others.^1,2^ These materials are categorized as salts composed merely by the combinations of anionic and cationic species with a typical melting temperature below 100 °C.^3^ Additionally, ILs are known by their exceptional physicochemical properties, including high ionic conductivity (10^–2^–10^–3^ S·cm^–1^), thermal stability, wide electrochemical windows, and negligible volatility.^4–6^ Due to the several combinations of anions and cations, it is possible to tailor IL properties including electrochemical stability and ionic conductivity. In addition, this tailoring capability allows the tuning of the ILs’ response to external stimuli, namely, magnetic and electric fields, heat, and light.^7,8^ .^6^

By incorporating ILs into a polymeric matrix, a new form of stimuli responsive, multifunctional, and flexible platform is developed.^1^ Their ability to form stable, flexible, and multifunctional polymer composites has opened new possibilities for advanced applications in electrochemical devices, membranes, and sensors and actuators.^9,10^ Among the many polymers used, electroactive polymers (EAPs) have proven to be suitable due to their tailorable and active response, and applicability in different areas such as sensors and actuators,^7,11^ energy harvesting,^12,13^ and biomedicine.^14,15^ Combining the common properties of most polymers, like cost effectiveness, easy processability, lightweight, and flexibility with a high dielectric constant, polarity, and electrochemical response, EAPs’ response can be additionally tailored by the addition of fillers, including ILs.^16^

Poly­(vinylidene fluoride) (PVDF) and its copolymers are EAPs that possess high chemical stability, ionic conductivity, and polarity. In addition, they can be easily processed in different forms and shapes including spheres, films, fibers, or membranes.^17^

As a biocompatible, nontoxic, and semicrystalline polymer with high polymorphism, PVDF can crystallize in different phases, depending on the processing conditions, with α, β, and γ being the most commonly implemented in advanced applications.^18^

Different works have demonstrated the synergy of combining PVDF with ILs and nanofillers, leading to enhanced dielectric, piezoelectric, and ionic conductivity characteristics. For instance, Sahrash et al.^19^ demonstrated the versatility of PVDF ionogels for electrochemical devices and membrane separation, showing that the incorporation of ILs led to improved ionic mobility, mechanical flexibility, and thermal stability. Correia et al.^1,11,20,21^ investigated how IL type and concentration influence polar β-phase formation and ionic conductivity in PVDF and its copolymers, leading to improved electromechanical response in sensors and actuators. Fernandes et al.^7,22,23^ developed PVDF-based IL composites for sensing applications, including humidity and pressure sensors, as well as actuators, where the presence of ILs enhanced stimuli sensitivity, β-phase formation, and ionic conductivity. In the biomedical field, Meira et al.^14^ used PVDF-based IL composites to obtain soft and flexible composites, with potential for artificial muscles and drug delivery systems.

Often in the development of IL/polymer composites, the leakage of the IL from the polymeric matrix occurs over time,^24,25^ preventing actual application beyond the proof of concept. The immobilization of the ILs in the matrix using solid supports like metal–organic frameworks (MOFs) presents itself as a possible solution.^19,26^ MOFs, also known as porous coordination polymers (PCPs), are microporous materials based on metal ions or clusters coordinated to organic ligands, allowing to tailor their physical-chemical properties and structure according to the aimed specific functional characteristics.^27^ They are known for their high porosity and surface area, being suitable for added functionalities depending on the nature of the metal components, organic linkers, or both.^28^ Macedo et al.^29^ developed a ternary polymer/MOF/IL composites based on polyvinylidene fluoride-*co*-hexafluoropropylene (PVDF-HFP), a PVDF copolymer, with MOF-808 and 1-butyl-3-methylimidazolium thiocyanate ([BMIM]­[SCN]). The composite demonstrated improved mechanical strength and ionic conductivity and is suitable for solid polymer electrolyte (SPE) applications.

The potential of polymer composites based on ILs and MOFs has already been shown in different areas,^30^ mainly in gas adsorption^31^ and separation, liquid-phase adsorption and separation, catalysis, and electrochemical devices.^32–36^

Besides the efforts dedicated to developing IL/MOF-based polymer composites, new insights into structural information are needed. Currently, most studies have focused on traditional characterization techniques such as nuclear magnetic resonance (NMR), X-ray diffraction analysis (XRD), and Fourier transform infrared spectroscopy (FTIR), which provide information on key aspects like IL–MOF interactions, IL and MOF interactions with the polymer matrix, phase, and crystal structures. However, these techniques do not allow for a precise understanding of the IL distribution within IL/MOF-based polymer composites and there is a lack of understanding regarding how the nanoscale spatial distribution and interaction of ILs and MOFs within a polymer matrix influence the resulting composite properties.^30^ In particular, the structural organization of ILs in MOF-containing PVDF composites and their direct effect on β-phase formation, crystallinity, and ionic conductivity have not been properly explored.

Therefore, this study is based on the hypothesis that the anion type of imidazolium-based ILs plays a critical role in determining both the structural integrity of the MOF and the nanostructured organization within the PVDF matrix. To the best of our knowledge, their effect is not well established.

In this work, PVDF ternary composites with the MOF HKUST-1 (20 wt %) and the ILs (20 wt %) 1-butyl-3-methylimidazolium thiocyanate ([Bmim]­[SCN]), 1-butyl-3-methylimidazolium dicyanamide ([Bmim]­[N­(CN)_2_]), and 1-butyl-3-methylimidazolium tetrachloroferrate ([Bmim]­[FeCl_4_]) were produced by solvent casting, thus studying the anion effect on the composite properties. The composites were characterized in terms of their morphological, physicochemical, thermal, and electrical properties. To complement these analyses and gain insight into nanoscale structural organization, small-angle neutron and X-ray scattering (SANS/SAXS) was employed. This multiscale approach enables the identification of key structure–property relationships relevant to the design of high-performance composites for sensing and energy storage applications.

## 2 Experimental Section

### 2.1 Materials

The polymer PVDF6010 (Mw 300–330 kDa) and the solvent *N*,*N*-dimethylformamide (DMF, ≥99.8% purity) were purchased from Solvay and Honeywell, respectively. The ILs, [Bmim]­[FeCl_4_], [Bmim]­[SCN], [Bmim]­[N­(CN)_2_], and HKUST-1 (Basolite C300) were purchased from Iolitec and Sigma-Aldrich, respectively. Some relevant characteristics of the IL and MOF are presented in Table 1.

**Table 1:**
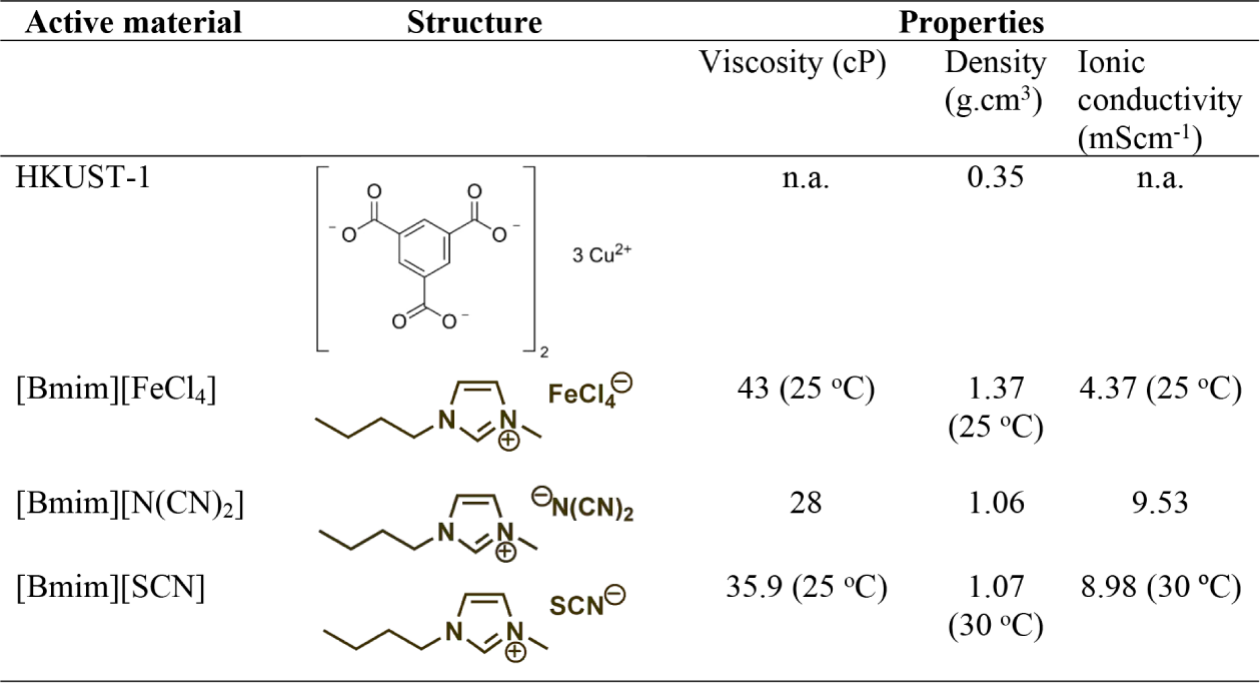
Characteristics of HKUST and Different ILs^a^

a Source from the provider (Sigma-Aldrich and IoLiTec).

### 2.2 Methods

For the preparation of the pristine polymer, 15 wt % of PVDF was dissolved in DMF in a ratio 15/85 wt %. After complete polymer dissolution, the solution was spread onto a glass substrate, and solvent evaporation was carried out at 210 °C for 10 min.^17^ This temperature was selected with the aim to ensure complete solvent removal and to study the effect of different IL types and MOF incorporation on PVDF characteristics.

The composite films were produced as follows: For the bicomposites, 20 wt % of MOF (HKUST-1) was dispersed in DMF using an ultrasonic bath (digital ultrasonic cleaner MODEL: ATM40-3LCD).For the ternary composites (IL/MOF/PVDF), 20 wt % of IL and 20 wt % of MOF were dispersed in DMF, also under an ultrasonic bath. Afterward, PVDF (PVDF/DMF 15/85 wt %) was added to the solution, and magnetic or mechanical agitation (for [Bmim]­[FeCl_4_], due to its magnetic nature) was applied until complete polymer dissolution. The final solution was spread onto a glass substrate, and solvent evaporation was carried out at 210 °C for 10 min. The solvent evaporation was conducted at 210 °C to ensure uniform film formation while maintaining the stability of the incorporated IL and MOF.^22^ After the mixture was cooled to room temperature, films with an average thickness of 44 μm were obtained.

### 2.3 Characterization

The morphology of the composites was analyzed by scanning electron microscopy (SEM), Hitachi S-4800, with an accelerating voltage of 5 kV. Previous to the measurements, the samples were coated with a thin gold layer by sputtering (Polaron SC502 device).

The crystalline phase of PVDF in the composite samples was evaluated by Fourier transform infrared spectroscopy in attenuated total reflectance mode (Jasco FT/IR-6100 equipment) (FTIR-ATR). Measurements were carried out at room temperature, after 64 scans with a resolution of 4 cm^–1^ in the 4000 to 600 cm^–1^ spectral range. The calculation of the crystalline electroactive β-phase fraction (*F*(β)) was obtained applying eq 1:

1 $$F\left(\beta \right)=\frac{A_{\beta }}{\left(\frac{K_{\beta }}{K_{\alpha }}\right)A_{\alpha }+A_{\beta }}$$

where *A*_*α*_ and *A*_*β*_ are the absorbances at 766 and 840 cm^–1^, corresponding to the α and β phases of the polymer, respectively, and *K*_*α*_ and *K*_*β*_ are the corresponding α and β absorption coefficients: 6.1 × 10^4^ and 7.7 × 10^4^ cm^2^·mol^–1^.^11,18^

Differential scanning calorimetry (DSC) measurements were performed under a N_2_ atmosphere to analyze the thermal behavior of the films. Measurements were carried out at a heating rate of 10 °C·min^–1^ in the temperature range from 25 to 200 °C (PerkinElmer DSC 6000 apparatus). The crystallinity degree (*X*_c_) was calculated after eq 2:

2 $$X_{c}=\frac{\Delta H}{x\Delta H_{\alpha }+y\Delta H_{\beta }}$$

with Δ*H* being the sample’s melting enthalpy, Δ*H*_α_ and Δ*H*_β_ the melting enthalpies corresponding to the α (93.07 J·g^–1^) and β phases (103.4 J·g^–1^) of PVDF, respectively, and *x* and *y* are the α and β phase proportions of each sample.^11,18^

To study the thermal degradation of the composite films, thermogravimetric analysis (TGA) was carried out under a synthetic air atmosphere over a temperature range from 40 to 800 °C and a heating rate of 10 °C·min^–1^ (NETZSCH STA 449F3).

The volumetric electrical conductivity (σ) of the samples was calculated from the slope of the current–voltage (*I*–*V*) curves measured in a Keithley 487 picoammeter/voltage source by applying voltages ranging from −10 to 10 V. Prior to the measurements, the samples were coated by magnetron sputtering (Edwards Scancoat Six Pirani 501) on both sides with 5 mm diameter gold electrodes. σ was obtained from the slope of the *I*–*V* curves as (eq 3):

3 $$\sigma =\frac{L}{RA}$$

where *R* is the electrical resistance, and *L* and *A* are the sample thickness and electrode area, respectively.

Small-angle neutron scattering (SANS) measurements, RB2220418 (doi.org/10.5286/ISIS.E.RB2220418), were performed using the SANS2D instrument at the ISIS Pulsed Neutron and Muon Source (Oxfordshire, UK).^37^ The instrument was operated in time-of-flight mode, using a wavelength band from 1.75 to 12.5 Å and two gas-tube detectors to provide a simultaneous merged *q*-range of 0.002 ≤ *q* (Å^–1^) ≤ 1.0. The main detector was placed at 12 m from the sample and the size of the beam was 8 mm. Measurements were performed at room temperature. The films were contained in thin aluminum foil envelopes and were attached to an enclosed automatic multiposition sample changer thermostated by a circulating fluid bath. The data were calibrated to an absolute scale by reference to the scattering from a partially deuterated polystyrene polymer blend.^38^

Small-angle X-ray scattering (SAXS) measurements were carried out with a Cu K_α_ radiation SAXSpoint 2.0 instrument (Anton Paar, Austria) with a hybrid photon-counting 2D EIGER R series detector (*q*-range: 0.07–5 nm^–1^). The measurements were carried out on a special plate for solid films.

## 3 Results and Discussion

### 3.1 Morphology

The cross-sectional SEM images of the PVDF-based composite containing the MOF HKUST-1 and the different ILs are presented in Figure 1.

**Figure 1:**
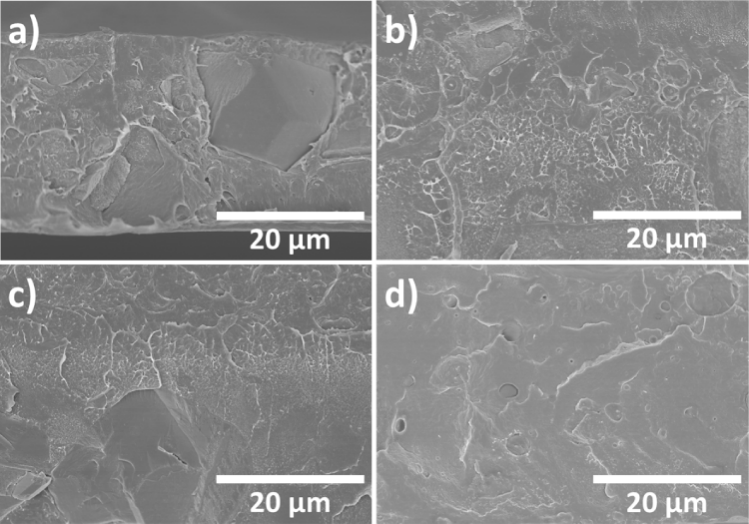
(a) PVDF + HKUST-1; (b) PVDF + HKUST-1 + [Bmim]­[FeCl_4_]; (c) PVDF + HKUST-1 + [Bmim]­[N­(CN)_2_]; (d) PVDF + HKUST-1 + [Bmim]­[SCN].

At the used processing conditions, with solvent evaporation performed at 210 °C, neat PVDF films are obtained in a compact microstructure, as no phase separation process occurs.^23^ With the MOF incorporation, as shown in Figure 1a, the PVDF/HKUST-1 composite presents the same compact microstructure of pristine PVDF. Furthermore, the presence of agglomerates with a maximum size of ∼17 μm of HKUST, dispersed within the polymer. The compact microstructure of the films also remained unchanged in IL/PVDF composites comprising the IL [Bmim]­[FeCl_4_].^39^ However, the incorporation of the ILs [Bmim]­[N­(CN)_2_] and [Bmim]­[SCN] allows a morphology with the typical spherulitic microstructure of PVDF.^40^

The inclusion of the ILs and the MOF in the ternary composites (Figure 1b–d) induces a thickness increase while maintaining the compact structure and a good dispersion of the fillers, as no agglomerates are observed. This indicates that the presence of ILs in the ternary composites probably leads to a destruction of the MOF structure as well as large-size inhomogeneities, indicating IL accumulation with parts of the destroyed MOF as well as IL agglomeration through the matrix. Thus, the agglomerates observed in PVDF/HKUST are not observed in IL/PVDF composites

### 3.2 Physical-Chemical Properties and Thermal Characterization

The physical-chemical and thermal properties of both PVDF/HKUST and the ternary composites PVDF/HKUST/IL for the different IL types are shown in Figure 2. Figure 2 also shows the influence of HKUST and IL on the electroactive phase and degree of crystallinity.

**Figure 2:**
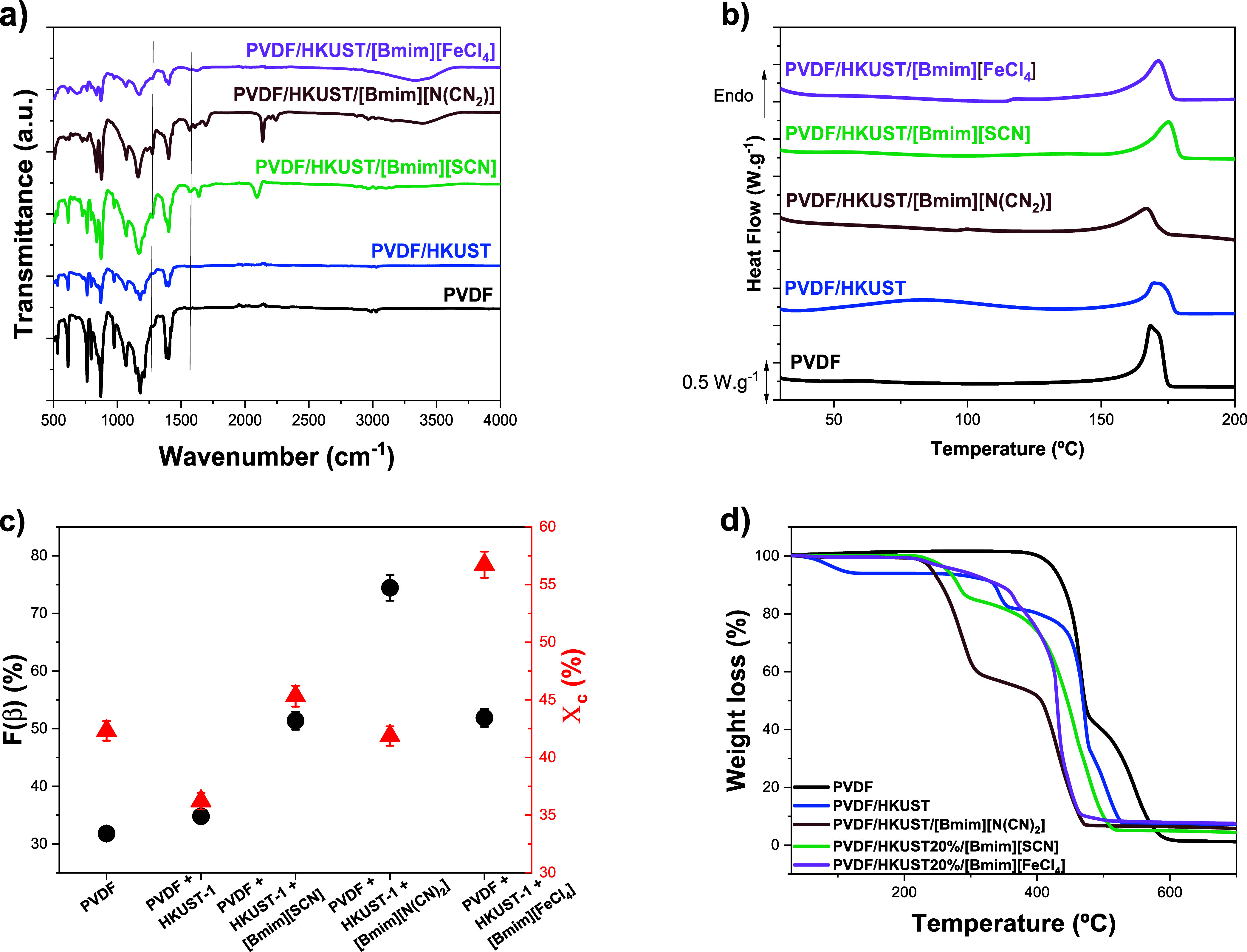
Physical-chemical characterization of PVDF, HKUST and PVDF + HKUST-1+[Bmim]­[FeCl_4_], PVDF+HKUST-1+[Bmim]­[N­(CN)_2_] and PVDF+HKUST-1+ [Bmim]­[SCN] samples: a) FTIR-ATR spectra; b) DSC themograms; c) β-phase content and degree of crystallinity; and d) TGA curves.

The vibration spectra of the films are presented in Figure 2a and the corresponding crystalline β-phase content, calculated by using eq 1, is shown in Figure 2c. The FTIR spectra of all samples (Figure 2a) show the typical absorption bands corresponding to the α- (766, 795, and 976 cm^–1^) and β-phases (840 and 1275 cm^–1^) of PVDF.^18^ As for the bands corresponding to the HKUST-1 MOF, the majority of the bands overlap with the PVDF polymer; however, the asymmetric stretching of the carboxylate group of Cu-BTC is visible at 1645 cm^–1^. In relation to the ILs, the presence of [Bmim]­[FeCl_4_] is confirmed by the peaks of the [Bmim]^+^ cations attributed to C–C stretching vibrations at 1564 cm^–1^.^7,41^ The presence of the ILs [Bmim]­[N­(CN)_2_] and [Bmim]­[SCN] is proven, respectively, by the presence of the peaks at 2164 cm^–1^ corresponding to the C–H stretching vibration of [N­(CN)_2_]^−^ and by the C–N vibration mode of the [SCN]^−^ anion at 2054 cm^–1^.^11^ Regarding the β-phase content (Figure 2c) calculated after applying eq 1 and presented in Table 2, the addition of H-KUST to the PVDF matrix does not induce significant differences in the amount of β-phase being 32% and 35% for the pristine polymer and the polymer composite, respectively. With the IL incorporation, there is evidence of an increase in the electroactive β phase, with the highest content observed for the ternary composite containing the IL [Bmim]­[N­(CN)_2_], reaching approximately 74%. This increase is attributed to the electrostatic interactions between the anions of the IL and the positive side of the PVDF dipoles,^20^ thereby promoting the orientation of the polymer chains in the all-trans chain conformation characteristic of the β-phase. Such electrostatic interactions facilitate the crystallization of the polymer in the phase with the highest dielectric constant and electroactive response of PVDF. The highest electroactive phase content observed for the PVDF/HKUST/[Bmim]­[N­(CN)_2_] composite also indicates that the interionic interactions within the HKUST MOF are favored by the [Bmim]­[SCN] and [Bmim]­[FeCl_4_] ILs by strengthening the hydrogen bonding between cation and anion, comparatively to the [Bmim]­[N­(CN)_2_] IL.^42^ Thus, while H-KUST induces the PVDF crystallization mainly in the α form crystalline phase, the incorporation of the different types of ILs promotes electroactive β phase formation.

**Table 2:** β-Phase and Degree of Crystallinity of the Different Samples

Sample	β-phase (%) (±3%)	Crystallinity (%) (±2%)
PVDF	32	42
PVDF/HKUST-1	35	36
PVDF/HKUST-1/[Bmim][SCN]	51	45
PVDF/HKUST-1/[Bmim][N(CN)_2_]	74	48
PVDF/HKUST-1/[Bmim][FeCl_4_]	52	57

The thermal transitions and degree of crystallinity of the samples were evaluated after the DSC measurements, as presented in Figure 2b. All composite samples present an endothermic peak between 150 and 180 °C, corresponding to the melting of the PVDF polymer.^11,18^ For the ternary composite sample with the IL [Bmim]­[N­(CN)_2_] the melting temperature occurs at slightly lower temperatures due to the crystalline phase destabilization resulting from the electrostatic interactions between the IL and the polymeric PVDF matrix.^11^ On the other hand, for the composite sample with the IL [Bmim]­[SCN] the melting temperature peak occurs at a somewhat higher temperature indicating stronger interaction between the HKUST and IL.

The degree of crystallinity of the samples was obtained after eq 2 (Figure 2c) and is presented in Table 2. The incorporation of HKUST into the PVDF matrix leads to a decrease in the crystallinity degree from 42% to 36%, as the MOF acts as a defect during polymer crystallization.^43^ Upon the addition of the different ILs to the PVDF-HKUST matrix, the degree of crystallinity increases, being independent of the IL type. The more noticeable increase is observed for the sample incorporating the [Bmim]­[FeCl_4_] IL (57%). This tendency to the crystallinity degree increase is not commonly observed for PVDF/IL composites, in which the crystallinity tends to decrease as a result of the plasticizing effect resulting from the IL-PVDF molecular chain interactions, indicating the relevance of the H-KUST–IL interaction.^20,21^ This suggests that the presence of HKUST-1 in the PVDF matrix plays a key role in mediating the effect of the ILs. It is likely that the HKUST–IL interactions promote the formation of an ordered microenvironment around the MOF particles, which facilitates PVDF chain alignment and nucleation, thereby counteracting the usual plasticizing effect of ILs in the PVDF matrix. The crystallinity degree values are also corroborated from the XRD measurements presented in Figure 3, which were also performed to evaluate potential structural changes in the PVDF/MOF/IL composite samples.

**Figure 3:**
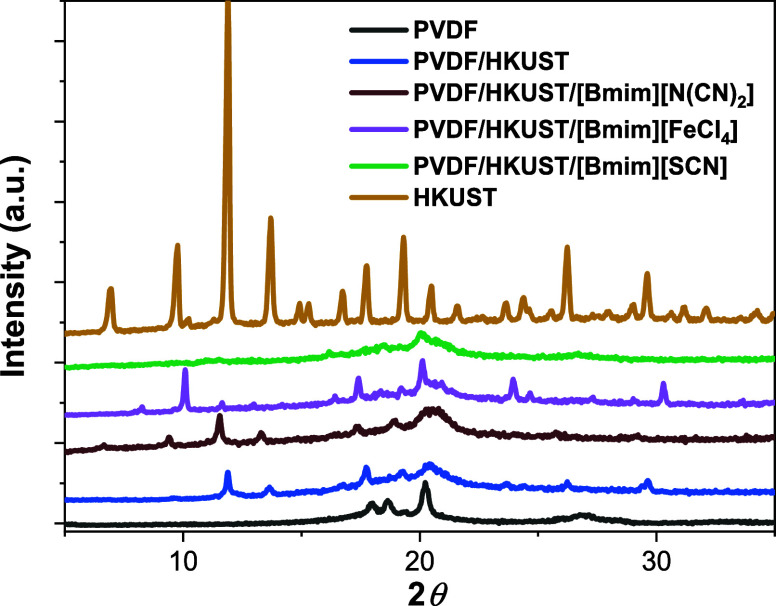
X-ray diffraction patterns of PVDF, HKUST and PVDF + HKUST-1 + [Bmim]­[FeCl_4_]; PVDF + HKUST-1 + [Bmim]­[N­(CN)_2_]; and PVDF + HKUST-1 + [Bmim]­[SCN] samples.

The influence of both IL and MOF on the PVDF thermal stability was evaluated by TGA measurements (Figure 2d). As observed, neat PVDF presents a first degradation step at approximately 418 °C corresponding to the scission of carbon–hydrogen (C–H) and carbon–fluorine (C–F) of PVDF.^44^ The composite PVDF-HKUST presents three degradation steps, occurring the first between 35 and 100 °C corresponding to the evaporation of residual solvent.^45^ The H-KUST degradation occurs at approximately 330 °C,^45^ followed by the PVDF degradation at 440 °C, occurring a slight increase in the thermal stability. In ternary composites incorporating the different ILs, two degradation steps are observed, presenting similar degradation temperatures of the different ILs, starting at 224 °C for the composites incorporating [Bmim]­[FeCl_4_] and [Bmim]­[N­(CN)_2_] and 244 °C for the composite incorporating [Bmim]­[SCN]. Independently of the anion type, the decrease of the thermal stability of the composites is ascribed to the catalytic influence of the IL’s anions and cations on the PVDF degradation reaction.

The crystal structure of the composites was thus analyzed based on the XRD peaks of PVDF (Figure 3). PVDF exhibits diffraction peaks at 2θ values of 18.63°, 20.30°, and 26.57°, where the first peak corresponds to the α phase (020), while the latter two are associated with the β crystalline phase (110) and (021), respectively.^46^ The characteristic diffraction peaks of HKUST-1, corresponding to its cubic crystal structure with space group (*Fm*3̅*m*) are also identified. Furthermore, in the PVDF/HKUST composite, several of these peaks remain visible at 2θ values of 9.77°, 11.86°, 13.64°, 17.75°, 19.32°, 23.64°, 26.22°, and 29.61°, corresponding to the (220), (222), (400), (511), (440), (444), (622), and (751) planes, respectively.^47^

Furthermore, it is observed in Figure 3 that by adding IL to PVDF samples with HKUST, the peaks corresponding to the MOF decrease in intensity due to the different interactions between IL and MOFs and their effect in the MOF structure. As we will discuss in the following, indicating that the incorporation of ILs can promote the destruction of the crystalline structure of the MOF with the IL, being accumulated with parts of the destroyed MOF, or contributing to IL agglomeration through the matrix.

### 3.3 Electrical Characterization

The electrical properties of the composites were analyzed after the *I*–*V* curves, from which the electrical conductivity was obtained using eq 3. The results are shown in Figure 4.

**Figure 4:**
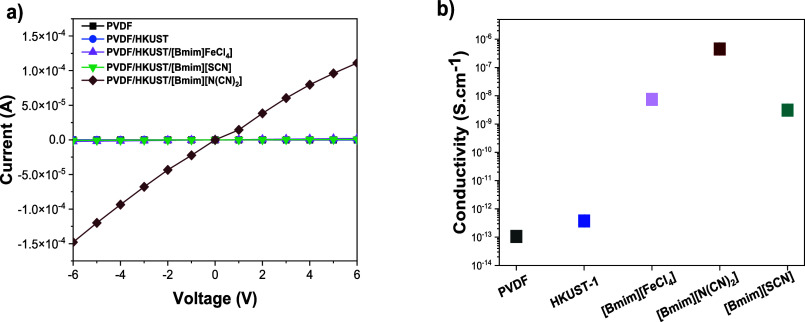
(a) *I*–*V* curves and (b) electrical conductivity values of PVDF, HKUST and PVDF + HKUST-1+[Bmim]­[FeCl_4_], PVDF+HKUST-1+[Bmim]­[N­(CN)_2_] and PVDF+HKUST-1+ [Bmim]­[SCN] samples.

All samples show a linear Ohmic response regarding the electrical properties. The addition of the MOF HKUST1 to PVDF induces a slight increase in the electrical conductivity from 1.06 × 10^–13^ to 3.74 × 10^–13^ S·cm^–1^, which can be attributed to the diffusion and adsorption of the water molecules from the air into the active sites of HKUST-1.^48^ However, this increase is less pronounced compared to the effect observed with the incorporation of ILs, where an increase in the ionic conductivity is noticeable.^49^ The addition of ILs, however, has a strong impact on the electrical conductivity with respect to the pristine PVDF and PVDF/HKUST, increasing to 7.48 × 10^–9^, 7.52 × 10^–7^, and 3.11 × 10^–9^ S·cm^–1^ for the ILs [Bmim]­[FeCl_4_], [Bmim]­[N­(CN)_2_] and [Bmim]­[SCN] IL containing composites, respectively. The increase in electrical conductivity is entirely attributed to the ionic characteristics of the ILs, which introduce a large number of charge carriers within the samples and enhance their mobility. The higher value for the ternary composite with the IL [Bmim]­[N­(CN)_2_] can also be related to the higher β-phase content and lower crystallinity, as well as the higher intrinsic ionic conductivity of [Bmim]­[N­(CN)_2_] when compared to the other ILs (Table 1).^11^ Further, the observed differences in the ionic conductivity values can be related to the size and structure of the anions, which influence the ion transport properties within the composite. Between the different types of ILs, the anion from the [Bmim]­[N­(CN)_2_] is smaller and more flexible compared to [FeCl_4_]^−^ and [SCN]^−^, allowing for more efficient charge transport and reduced steric hindrance. Additionally, the [FeCl_4_]^−^ anion is larger and more complex comparatively to the other anion types, which contributes to higher viscosity and lower ionic mobility as a result of a more hindered ion movement, thereby reducing the overall conductivity. Similarly, the [SCN]^−^ anion (smaller than [FeCl_4_]^−^) exhibits lower conductivity compared to [N­(CN)_2_]^−^ due to its comparatively lower intrinsic ionic conductivity and less effective interaction with the polymer matrix. Therefore, the enhanced conductivity of the PVDF/HKUST/[Bmim]­[N­(CN)_2_] composite arises from a combination of factors, including increased polar β-phase content, reduced crystallinity, lower viscosity, and the intrinsically higher ionic conductivity of [Bmim]­[N­(CN)_2_], promoting greater charge carrier availability and improved ion mobility within the material. Further, the observed higher ionic conductivity can also be indicative that [Bmim]­[N­(CN)_2_] does not enter into the HKUST cavities.

### 3.4 Structural Organization of the IL and MOF within the PVDF Matrix

SANS and SAXS measurements were conducted to gain a deeper understanding of the internal morphology of the samples on the nanometric scale (Figure 5a–d). Starting with the shape of all curves, their smoothness is a typical scenario from polydisperse systems.^50^ Regarding the scattering curves for the neat PVDF sample, they exhibit a characteristic behavior representative of semicrystalline polymer materials that are heterogeneous at the submicron size range. This behavior can be detected in all curves since all contain PVDF. Interestingly, different film compositions result in distinct SANS curves, indicating the presence of diverse nanoscale structures within the films. All curves were fitted using SasView software (SasView Version 5.0.6, Zenodo (n.d.)) using different fitting models, and the resulting data are summarized in Tables 3 and 4. It is important to notice that the SANS features are visible only when there is enough scattering length density (SLD) contrast between the components (SLD difference of at least ∼1 cm^–2^). In our case, the SLD for air, PVDF, MOF, [Bmim]­[N­(CN)_2_], [Bmim]­[SCN], and [Bmim]­[FeCl_4_] is 0 × 10^10^, 2.50 × 10^10^, 2.30 × 10^10^, 1.78 × 10^10^, 1.13 × 10^10^, and 1.56 × 10^10^ cm^–2^, respectively.

**Table 3:** SANS Fitting Parameters: Peak Position, Porod Exponent, *R*_g_, and Power Law from Regions I and II

Sample	Peak position (nm^–1^)	Porod exponent
**PVDF**	0.473 ± 0.008	3.8 ± 0.2

Sample	R_g_ I (nm)	q^‑P^ I	R_g_ II (nm)	q^‑P^ II
**PVDF/HKUST 20%**	44 ± 1	3.5 ± 0.1	8 ± 1	3.0 ± 0.2
**PVDF/HKUST/[Bmim][N(CN)**_**2**_ **]**20/20**%**	60 ± 1	2.357 ± 0.007	8.1 ± 0.2	3.40 ± 0.02
**PVDF/HKUST/[Bmim][SCN]**20/20**%**	148 ± 69	3.2 ± 0.2	6.7 ± 0.5	3.4 ± 0.6
**PVDF/HKUST/[Bmim][FeCl**_**4**_ **]**20/20**%**	78 ± 8	3.3 ± 0.1	8.4 ± 0.3	3.1 ± 0.2

**Table 4:** SAXS Fitting Parameters: Peak Position, Porod Exponent, *R*_g_, and Power Law from Regions I and II

Sample	Peak position (nm^–1^)	Porod exponent
**PVDF**	0.4873 ± 0.0003	4.25 ± 0.03
**PVDF/HKUST/[Bmim][SCN]**20/20**%**	0.4869 ± 0.0007	3.549 ± 0.008
**PVDF/HKUST/[Bmim][FeCl**_**4**_ **]**20/20**%**	0.3783 ± 0.0004	3.7818 ± 0.0003

Sample	Rg I (nm)	q^‑P^ I	Rg II (nm)	q^‑P^ II
**PVDF/HKUST 20%**	29.5 ± 0.3	3.92 ± 0.04	8.6 ± 0.1	1.92 ± 0.01
**PVDF/HKUST/[Bmim][N(CN)**_**2**_ **]**20/20**%**	33 ± 12	3.4 ± 0.5	7.60 ± 0.05	2.59 ± 0.04

**Figure 5:**
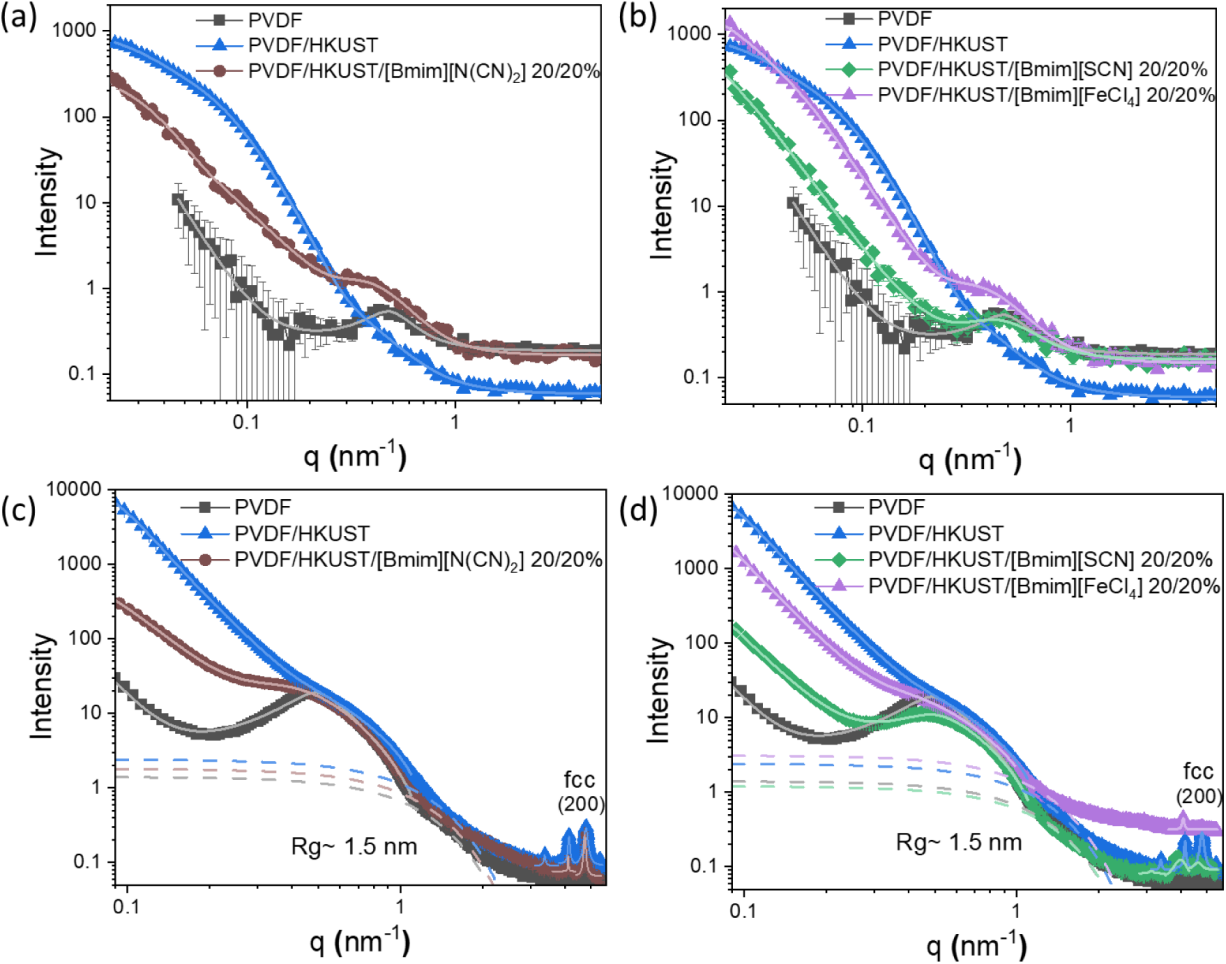
SANS (a,b) and SAXS (c,d) curves of the samples. The full and dashed lines represent the curve’s fittings. FCC means the face-centered cubic crystalline structure of the MOF.

The – incorporation of H-KUST MOF into PVDF leads to a new inhomogeneity signal in the curves at the low *q*-region, named Region I (Figure 6a,b), which is assumed to be related with the pore space created between the interface of MOF agglomerates and it correlates well with previous works for polymer films with MOFs.^51,52^

**Figure 6:**
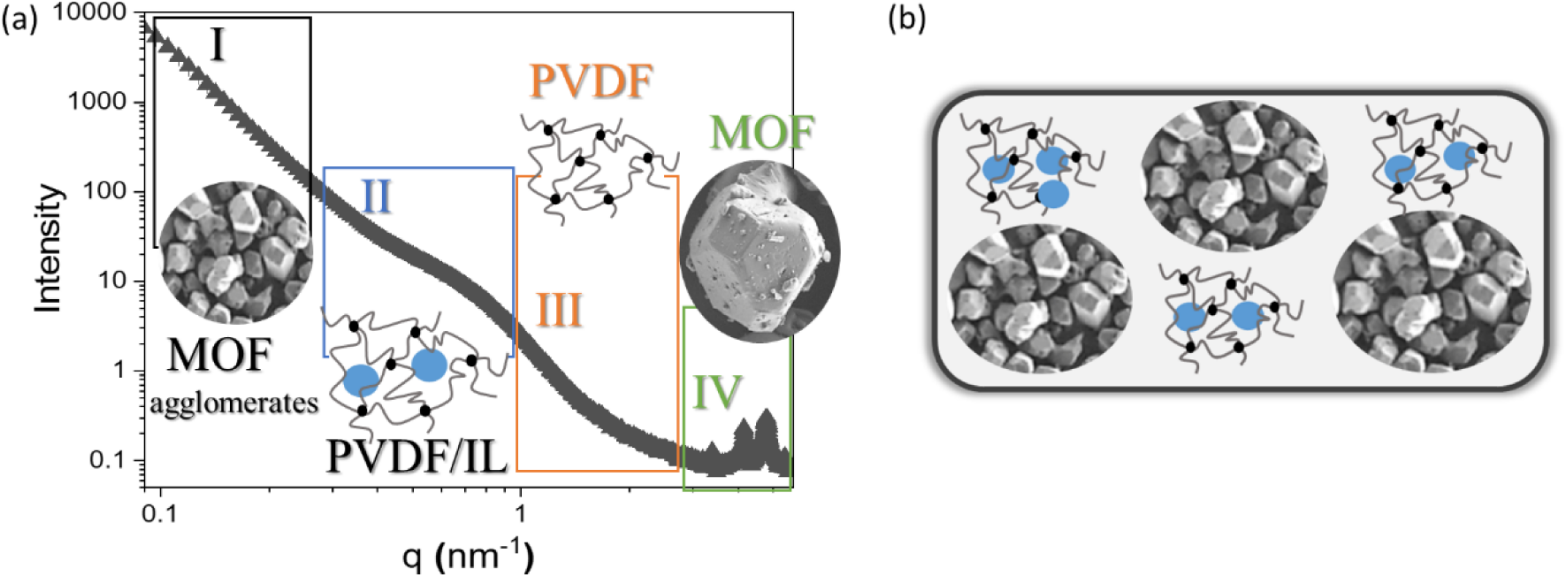
General scheme of the different regions identified from the SANS and SAXS curves (a) and their respective illustrative representation (b). The size of inhomogeneities in each region is as follows : Region I: >100 nm; Region II: 30 nm; Region III: 2 nm; and Region IV: 0.1 nm.

Region I, which can be better observed from SANS data (due to the lower minimum *q* values from SANS, *q*_min_ = 0.02 nm^–1^, when compared to SAXS, *q*_min_ = 0.1 nm^–1^) is related to a Guinier regime followed by power-law decay showing inhomogeneities with sizes between *R*_g_ = 60–150 nm with a surface fractal distribution (*q* ^–P^, *p* > 3). In the case of the PVDF/HKUST/[Bmim]­[N­(CN)_2_] sample, the addition of IL to the PVDF/HKUST composites leads to a shift of this region to lower *q* values when compared to HKUST/PVDF samples without IL, meaning that we are in the presence of larger size inhomogeneities, still with surface fractal distribution. We assume that these inhomogeneities are related to part of the IL that did not enter the MOF’s pores and stayed accumulated at the surface of the MOF’s agglomerates, increasing therefore the size of the space created between agglomerates. In the case of the incorporation of ILs [Bmim]­[SCN] and [Bmim]­[FeCl_4_] to the PVDF/MOF composites, this signal is less prominent from SANS/SAXS data due to the instrumental limit. Interestingly, the incorporation of these two ILs destroys the crystalline structure of the MOF (verified by SAXS and XRD data), thus we assume that the large size inhomogeneities are related with a mix of IL accumulated with parts of destroyed MOF or with IL agglomeration through the matrix.

With respect to Region II, which is better observed in the SAXS data, two distinct models were applied for the fitting (broad peak and Guinier followed by power law decay models). In the case of neat PVDF, a broad peak model was applied to fit the data, enabling the calculation of the scattering density length correlations for soft amorphous materials. The peak position (*q*_0_) is correlated with the *d*-spacing of phase-separated systems according to $q_{0}=2\pi /d_{0}$ *q*_0_ = 2π/*d*_0_, where *d*_0_ represents the scattering distance between crystals, lamellae, or clusters in the PVDF network.^53^ The obtained fitting for neat PVDF curves revealed a peak position of ∼0.47 nm^–1^ (distance ∼13 nm) with a Porod exponential of 3.8, revealing a surface fractal organization of the system, which is in good agreement with previously published data for PVDF-based polymer materials.^29,54,55^ The addition of MOF to the PVDF matrix does not produce changes in the position of the inhomogeneities in Region II (*R*_g_ ∼ 8 nm), although there is a decrease in the intensity of the peak, which is related to the PVDF’s lower crystallinity for PVDF/MOF samples, which is in good agreement with DSC/XRD results. On the other hand, the addition of ILs [Bmim]­[N­(CN)_2_], [Bmim]­[SCN], and [Bmim]­[FeCl_4_] into the MOF/PVDF matrix alters the peak positions, with the shift being particularly pronounced for [Bmim]­[N­(CN)_2_]. This shift corresponds to a change in the crystalline structure of PVDF, aligning well with the FTIR data, which indicates that the neat PVDF sample predominantly exhibits the α-phase. In contrast, the PVDF/MOF samples containing ILs primarily display the β-phase. In relation to the nature of these inhomogeneities spotted in Region II, it is assumed that it is the result of the interaction between the IL and the PVDF polymeric chains, which in the case of PVDF/MOF/[Bmim]­[SCN] and PVDF/MOF/[Bmim]­[FeCl_4_] samples present surface fractal organization and in the case of PVDF/MOF and PVDF/MOF/[Bmim]­[N­(CN)_2_] samples present mass fractal organization.

With respect to Region III, it is related to a small inhomogeneity visible across all samples but only visible from the SAXS curves. These inhomogeneities with a medium size of *R*_g_= 1.5 nm are related to the PVDF polymeric chain organization. SANS-SAXS techniques were successfully used to study ionic surfactants in hydrogels,^56^ MOF-IL composites,^37^ MOF-PVDF composites,^57^ and IL-PVDF composites.^58^ Recently, complex three-component systems are also being investigated,^59^ reporting results in line with the present ones.

Finally, in Region IV, the peaks localized at a *q* range >3 nm^–1^, fitted using the Lorentzian model, are related with the diffraction peaks from the MOF. The third peak corresponds to a real-space distance of 13.16 Å, and this *d*-spacing is consistent with the crystal structure of the MOF of around 13.17 Å (Miller indexes: 200), as extracted from the XRD data measured in these samples. This feature can only be visible for samples comprising MOF. However, for those samples where the addition of IL destroys the MOF (as it is the case of [Bmim]­[SCN] and [Bmim]­[FeCl_4_] ILs) probably as a result of their higher size, hindering ion movement and their interaction with the polymer matrix, it can be observed that the disappearance/shift of some of these peaks. As previously reported, both [Bmim]­[SCN] and [Bmim]­[FeCl_4_] ILs contain strongly coordinating anions, which, combined with their higher viscosities, hinder the diffusion of the ILs into the polymer matrix. This limited mobility leads to their accumulation at the MOF surface, promoting partial MOF degradation or disrupting its crystalline diffraction pattern. In contrast, [Bmim]­[N­(CN)_2_], containing a smaller and less coordinating anion as well as a lower viscosity, distributes more homogeneously within the MOF, thereby minimizing its disruptive effect on the MOF structure.

## 4 Conclusions

The present work reports the development of composites incorporating MOF (HKUST), distinct imidazolium ILs ([Bmim]­[FeCl_4_], [Bmim]­[N­(CN)_2_] and [Bmim]­[SCN]) sharing the same cation, and distinct anions with different anion sizes, within a polymer matrix. The objective is to study the PVDF/HKUST and PVDF/HKUST/IL interactions and structural organization in this type of ternary composites suitable for a wide variety of applications.

Independently of the filler type, all of the developed composites present a compact microstructure.

The physical-chemical characterization of the materials revealed no chemical changes in the PVDF chemical structure upon the incorporation of the MOF and IL. Regarding the electroactive β phase content, independently of the IL type, their incorporation results in an increase of the β-phase, with the highest amount observed in the ternary composite containing the IL [Bmim]­[N­(CN)_2_], reaching approximately 74% as a result of the electrostatic interactions between the anions of the IL and the positive side of the PVDF dipoles. Similarly, the crystallinity degree of the samples is also affected by the inclusion of the filler types, decreasing with the HKUST incorporation from 42% to 35% and increasing by incorporating the different ILs, with the sample incorporating [Bmim]­[FeCl_4_] IL displaying the highest crystallinity degree (57%).

The internal morphology of the samples at the nanometric scale was studied by SANS and SAXS revealing the presence of polydisperse systems. Among all the samples, the addition of [Bmim]­[SCN] and [Bmim]­[FeCl_4_] to the PVDF/MOF composites results in the destruction of the crystalline structure of the MOF, and large-sized structural inhomogeneities characterize the composites, related to a mix of IL accumulated with parts of destroyed MOF or with IL agglomeration through the matrix, indicating that ILs did not enter in the MOF pore space.

Thus, the present work establishes an essential contribution to the IL–HKUST interaction and its influence into the physical-chemical properties of the ternary polymer-based composite systems, important factors to take into account for their applicability in different fields, including sensors and energy storage systems.

Besides the strong improvements in the understanding of the IL-MOF-PVDF systems, the present work is limited to a specific MOF (HKUST) and a narrow set of imidazolium ILs. Thus, further studies involving different MOF structures and IL families are needed to generalize these findings and optimize the composites for targeted applications.
